# Undernutrition and dietary diversity score and associated factors among lactating mothers in Northwest Ethiopia

**DOI:** 10.3389/fnut.2024.1444894

**Published:** 2024-09-27

**Authors:** Mahider Awoke Belay

**Affiliations:** Department of Public Health, College of Medicine and Health Science, Injibara University, Injibara, Ethiopia

**Keywords:** lactating mothers, undernutrition, dietary diversity score, associated factors, Ethiopia

## Abstract

**Background:**

Maternal undernutrition negatively influences both maternal and child health, as well as economic and social development. Limited research has been conducted on both the nutritional status and dietary diversity score among lactating mothers. Therefore, the present study aimed to determine the magnitudes of undernutrition and dietary diversity scores and their associated factors among lactating mothers in Bahir Dar City, Northwest Ethiopia.

**Methods:**

A community-based cross-sectional study was conducted from March to May 2021. Systematic random sampling and interview-administered questionnaires were employed. Dietary diversity score and nutritional status were measured using a 24-h recall and body mass index (BMI), respectively. Data entry and analysis were performed using EpiData version 3.02 and SPSS version 24 software, respectively. Both the bivariable and multivariable binary logistic regression analyses were performed, and the strength of association was measured in terms of odds ratio.

**Results:**

The prevalence of undernutrition and low dietary diversity scores among respondents were 13.5% (95% CI; 10.4, 17.2) and 64.8% (95% CI, 60.0, 69.4), respectively. The significant factors for undernutrition were being young [AOR = 2.30, 95% CI (1.09, 5.43)], having low dietary diversity score [AOR = 2.26, 95% CI (1.01,5.10)], having poor nutritional knowledge [AOR = 2.56, 95% CI (1.03, 6.51)], meal frequency less or equal to 3 times per day [AOR = 4.06, 95% CI (0.71, 9.65)], educational status being primary school [AOR = 3.20, 95% CI (1.01, 9.11)], and educational status of husband being secondary school [AOR = 2.28, 95% CI (1.25, 8.53)]. Age between 20 and 30 years [AOR = 1.46, 95% CI (1.01, 2.48)], being food insecure [AOR = 3.41, 95% CI (1.21, 9.63)], and being poorest [AOR = 2.31, 95% CI (1.02, 5.32)] were associated with the dietary diversity score.

**Conclusion:**

A high prevalence of undernutrition and low dietary diversity scores were recorded in the current study area. Age, educational status of lactating mothers and their husbands, nutritional knowledge, dietary diversity, and meal frequency were significant factors associated with undernutrition. Age, food security, and wealth index were associated with the dietary diversity score.

## Introduction

Mothers are more likely to suffer from nutritional deficiencies during breastfeeding because of poor eating habits, physiological changes, and various sociodemographic factors (1). Inadequate nutritional intake before pregnancy, throughout pregnancy, and during breastfeeding increases postnatal nutritional stress and maternal health risk, resulting in a high maternal death rate (2, 3). Adequate energy consumption and a balanced diet that includes fruits, vegetables, and animal products throughout the lifecycle help ensure that women enter pregnancy and breastfeeding without deficits and acquire sufficient nutrients during periods of increased demand (4).

Dietary diversity (DD) is defined as the number of various food types ingested over a certain period to provide optimal intake of vital nutrients that can enhance health and physical and mental development (5). Food adequacy and nutritional quality during breastfeeding are critical for mother and child health (6). Good dietary diversity among lactating mothers has been linked to their children’s good health, growth, and development (7, 8).

Maternal undernutrition is highly prevalent in low-income and middle-income countries, resulting in substantial increases in mortality and overall disease burden (9). Approximately 240 million women in low- and middle-income countries (LMIC) experience undernutrition (BMI < 18.5) (10). The pooled prevalence of underweight among women in Sub-Saharan Africa (SSA) was 8.87% (11). The pooled prevalence of undernutrition and low dietary diversity scores among lactating mothers was 23.86 and 50.3%, respectively, in Ethiopia (12, 13).

Maternal undernutrition has a negative influence on maternal and child health, economic, and social development. Many women of reproductive age suffer from micronutrient deficiencies, which are associated with negative health outcomes (14–16). Women who are underweight or overweight before pregnancy have increased risk factors such as gestational diabetes, hypertension, preeclampsia, eclampsia, cesarean section, protracted labor, miscarriage, postpartum hemorrhage, anemia, hypothyroidism, tiredness, bleeding during birth, obstructed labor, morbidity, mortality, and poor pregnancy and breastfeeding outcomes (17, 18).

Malnutrition in women occurs at all phases of life due to poor quality food, inadequate services (lack of public needs such as health facilities, transport, and communication facilities), and a weak enabling environment. Several factors limit women’s access to proper diets and care, including limited availability and access to nutritious, safe, and affordable foods; limited opportunities for women to access nutrition services; limited knowledge of the importance of preconception nutrition care; and harmful gendered social norms and social and cultural practices (19–21). To reduce maternal mortality and increase optimum mother and child nutrition, the Cooperative for American Remittances to Europe (CARE) employs numerous interventions that are supported and encouraged by many techniques rather than relying on a single intervention (22). Sustainable Development Goal 2 aims to end hunger and malnutrition by 2030 (23). Providing a broad and appropriate diet may serve as the cornerstone of long-term sustainable measures to combat global malnutrition (5). According to the findings of several studies, lactating mothers have inadequate nutritional and dietary diversity (24–29).

Although we understand the causes of malnutrition, the prevalence and associated factors vary from setting to setting and over time. In addition to nutrition interventions, undernutrition among lactating mothers is a significant public health issue in developing nations. As a result, ongoing assessment of the prevalence and associated factors of undernutrition among lactating mothers is critical for prioritizing, designing, and launching intervention programs based on relevant data. Even though there is some evidence on the nutritional status, dietary diversity score, and associated factors among lactating mothers separately, there has been very little research in Ethiopia that has investigated both nutritional status and dietary diversity scores and their associated factors among lactating mothers.

Furthermore, no data are available on the nutritional status, dietary diversity score, and associated factors among lactating mothers in Bahir Dar, Ethiopia. Therefore, the current study aimed to establish the magnitudes of undernutrition and dietary diversity scores and associated factors among lactating mothers in Bahir Dar City, Northwest Ethiopia. This will aid in the development of appropriate interventions to increase maternal nutrition, as well as in the easy availability of information for future researchers.

## Methods and materials

### Study area and period

This study was conducted in Bahir Dar City, Northwest Ethiopia, the capital city of the Amhara region and 565 km from Addis Ababa, the capital city of Ethiopia. Based on the 2020 population report from the Bahir Dar City administrative office, there are 10,310 lactating mothers with children less than 2 years old in the city (30).

### Study design and population

A community-based cross-sectional study was conducted from March to May 2021. All lactating mothers whose children were 6 months to 2 years old in Bahir Dar City were considered the source population. Lactating mothers whose children were 6 months to 2 years old and who lived in the selected kebeles in Bahir Dar city were considered the study population.

### Inclusion and exclusion criteria

All lactating mothers who had children aged 6 months to 2 years and lived for a minimum of 6 months in Bahir Dar City were included in the study. Lactating mothers who had confirmed pregnancy and celebrated ceremonies within the last 24 hour were excluded from the study.

### Sample size determination and sampling techniques

The sample size was determined using a single population formula, $n=\frac{\left(Za/2\right)2\;P\left(1-P\right)}{d2}$ , the prevalence of underweight was 48.8% in a study conducted in central Ethiopia (31), with a 5% marginal error and a 95% confidence interval (CI), the sample size was calculated to be 384, and the final sample size was 423 after adding a 10% non-response rate. A systematic random sampling technique was used to select respondents. Eight kebeles were selected among 26 using a lottery method. The participants were allocated to each selected kebele using the population proportion to the size of each kebele. A simple random sampling technique was employed to select study participants from households with more than one respondent.

### Data collection tools and procedures

The data were collected through face-to-face structured interviews. After reviewing several related studies, an Amharic questionnaire, which was first written in English was updated to reflect local circumstances. This revision was carried out by 10 data collectors with a Bachelor of Science in Nursing, who were supervised by health officers with Bachelor’s degrees, after receiving 2 days of Amharic language training from the principal investigators on procedures of anthropometric measurement, interviewing technique, filling questionnaires, and respondent engagement. A standardized questionnaire was used to collect information on sociodemographic and economic factors. FANTA III created the Household Food Insecurity Access Scale (HFIAS), a standardized, and validated tool, to assess the level of household food insecurity (32).

The Household Food Insecurity Access Scale (HFIAS) includes nine occurrence items that show varying levels of food insecurity severity, along with nine “frequency-of-occurrence” follow-up questions that were asked over 4 weeks (past month) (33). To evaluate respondents’ nutritional knowledge, I asked 10 multiple-choice and yes/no questions covering the benefits of consuming different nutrients, sources of these nutrients, the need for extra meals during lactation, and the significance of iron and folic acid supplements. The total possible score for this nutritional knowledge assessment was 24 (34).

Dietary diversity was assessed using a qualitative recall of all food types consumed a day (24 h) before data collection. Ten food categories were used to calculate the dietary diversity score defined by FAO and FANTA III (35). The respondents’ nutrition knowledge was assessed by inquiring whether they had received any nutrition advice from a health professional. The household wealth index was used to measure respondents’ socioeconomic level by asking them about their household assets and housing features.

Anthropometric measurements were used to estimate the nutritional status of the respondents using the body mass index (BMI). Measurements were taken at the end of the interview using calibrated equipment and established procedures. This included determining the weight and height of lactating mothers. Mothers’ weights were measured to the nearest 100 g using a calibrated portable digital scale (the UNICEF electronic weighing scale) after they removed their shoes and put on light clothing. The respondent’s height was measured to the nearest 0.1 cm while they stood upright with their shoulders level, hands at their sides, and with their head, scapula, buttocks, and heels touching the base of a vertical measuring board equipped with a sliding head bar. The respondent responses were based on their willingness.

### Study variables

#### Dependent variables

The dependent variables include undernutrition and low dietary diversity scores.

#### Independent variables

The independent variables include age, child’s age, educational status of the lactating mother and her husband, religion, occupational status of the lactating mother and her husband, marital status, number of children, family size, wealth index, household food security status, meal frequency, access to nutrition information, food restrictions, and respondent’s nutritional knowledge.

### Operational definitions

*Undernutrition* is defined as a BMI of <18.5 kg/m2 (36).

*Minimum dietary diversity for women (MDD-W)* is defined using the 10 food groups recommended by the Food and Agriculture Organization (FAO) and the United States Agency for International Development (USAID) (37). The mother was asked about the types of food she consumed in the 24 h preceding the survey, regardless of the portion size.

The Minimum Dietary Diversity Score (MDDS) can be classified as follows:

*Low*: The mother consumed <5 food groups out of the 10.

*Adequate*: The mother consumed ≥5 food groups out of the 10.

*Household Food Insecurity Access Scale (HFIAS)* can be scored and classified as food secure or food insecure.

Food security includes persons in households where all members show no or minimal evidence of food insecurity (≤1 out of 27).

Food insecurity includes persons in households where all members feel anxious about running out of food or compromising on the quality of foods they eat by choosing less expensive options (2–27 out of 27).

Food restriction shows that limiting one’s food intake may have unanticipated implications for individuals who undertake it (38).

### Data management and analysis

The data were entered into EpiData version 3.02 software after being checked for completeness and consistency and then exported to SPSS version 24 for analysis. The wealth status of respondents was determined using principal component analysis, and eigenvalues were applied to compute the wealth status of respondents for each component. Descriptive analysis was performed using numerical summary measures, which were then reported in the form of texts, frequency tables, and figures. The lactating mother’s body mass index (BMI) was measured by dividing her weight in kilograms by her height in meters squared and classified as undernourished if her BMI is less than 18.5 kg/m2 (39).

Bivariable binary logistic regression analysis was performed to investigate the association between independent factors and dependent variables. The variables with a *p*-value of <0.25 in the bivariable analysis were incorporated into the multivariable analysis to identify independent predictors of undernutrition and low dietary diversity scores. Finally, factors with a *p*-value of <0.05 in the multivariable binary logistic regression analysis were substantially linked to undernutrition and low dietary diversity scores. The Hosmer–Lemeshow test was used to assess model fitness, and the level of association was calculated using the odds ratio.

### Data quality control

To ensure consistency, the data collection instrument produced in English was translated into Amharic and then returned to English. Initially, data quality was ensured by carefully designing the questionnaire and data gathering procedures. The investigator provided 2 days of training to data collectors and supervisors on instruments, data collection methods, anthropometric measurements, ethical considerations, and research objectives. Prior to data collection, 5% (*n* = 22) of the participants took a pretest. Every lactating mother’s height and weight were measured twice, and the average measurement was obtained. The proper functioning of digital weight scales was confirmed every time before the start of weight measurement, and the reading scale was ensured to be exactly zero by utilizing a portable reference weight of 1 kg. Two BSc health officers were in charge of supervision, and they constantly checked the data for completeness, correctness, and consistency during the data collection period. Overall, the investigator was available to answer questions and clarify any concerns raised by the data collectors and supervisors.

## Results

### The sociodemographic and socioeconomic status of the respondents

The median age of the respondents was 28.0 ± 6.0 (median ± IQR), with a response rate of 98.1%. Most respondents (41.9%) had college or above educational status. Approximately 82% of the lactating mothers were followers of the Orthodox religion. Approximately 43% of the respondent’s husbands were government employees. Regarding the wealth index, 75 (18.1%) and 126 (30.4%) respondents were the poorest and richest, respectively (Table 1).

**Table 1:** Sociodemographic and socioeconomic characteristics of lactating mothers in Northwest Ethiopia (*N* = 415).

Variables	Categories	Frequency	Percentage (%)
Age in years	20–29	244	58.8
	30–39	158	38.1
	40–45	13	3.1
Child’s age	6 months to 1 year	220	53.0
	1–2 years	195	47.0
Educational status of the lactating mother	Cannot read and write	52	12.5
	Primary school	93	22.4
	Secondary school	96	23.1
	College and above	174	41.9
Educational status of the husband	Cannot read and write	21	5.3
	Primary school	69	17.3
	Secondary school	73	18.3
	College and above	236	59.1
Religion of lactating mother	Orthodox	342	82.4
	Muslim	63	15.2
	Protestant	10	2.4
Occupational status of the lactating mother	Housewife	163	39.3
	Government employee	109	26.3
	Private employee	55	13.3
	Merchant	49	11.8
	Daily laborer	13	3.1
	Other	26	6.3
Occupational status of the husband	Merchant	87	21.9
	Government employee	169	42.5
	Private employee	98	24.6
	Daily laborer	17	4.3
	Other	27	6.8
Marital status of the lactating mother	Married	388	93.5
	Divorced	25	6.0
	Widowed	1	0.2
	Single	1	0.2
Number of children	Less than or equal to five(≤5)	411	99.0
	Greater than five(>5)	4	1.0
Family size	Less than or equal to five(≤5)	332	80.0
	Greater than five(>5)	83	20.0
Wealth index	Poorest	75	18.1
	Poor	97	23.4
	Middle	51	12.3
	Rich	66	15.9
	Richest	126	30.4

### Food security status, nutritional knowledge, and meal frequency of lactating mothers

Approximately 15% of respondents were food insecure. Regarding the nutritional knowledge of the respondents, 144 (34.7%) were poor. Most respondents ate three times per day (Table 2).

**Table 2:** Food security status, nutritional knowledge, meal frequency, dietary diversity, nutritional status, access to nutrition information, and food restrictions among lactating mothers in Northwest Ethiopia (*N* = 415).

Variables	Categories	Frequency	Percentage (%)
Food security status	Food secure	353	85.1
	Food insecure	62	14.9
Frequency of meals per day	2 or less times per day	43	10.4
	3 times per day	206	49.6
	4 times per day	122	29.4
	5 or more times per day	44	10.6
Nutritional knowledge status	Poor knowledge	144	34.7
	Medium knowledge	142	34.2
	Good knowledge	129	31.1
Dietary diversity scores	Low dietary diversity	269	64.8
	High dietary diversity	146	35.2
Nutritional status(BMI)	Malnourished(<18.5)	56	13.5
	Normal(18.5–24.9)	265	63.9
	Overweight(25.0–29.9)	82	19.8
	Obese(≥30)	12	2.9
Mid-upper arm circumference (MUAC)	Undernourished(<21 cm)	13	3.1
	Normal(≥21 cm)	402	96.9
Received nutrition information from health professionals	No	283	68.2
	Yes	132	31.8
Food restrictions in the community during lactation	No	388	93.5
	Yes	27	6.5

### Nutrition information and food restrictions for lactating mothers

Approximately 132 (32%) of the respondents received nutrition information from health professionals when they visited the health facility. Twenty-seven (6.5%) of the respondents said that there were food restrictions in the community during the lactation period (Table 2).

### Prevalence of undernutrition, low dietary diversity score, and food group consumption

**Figure 1:**
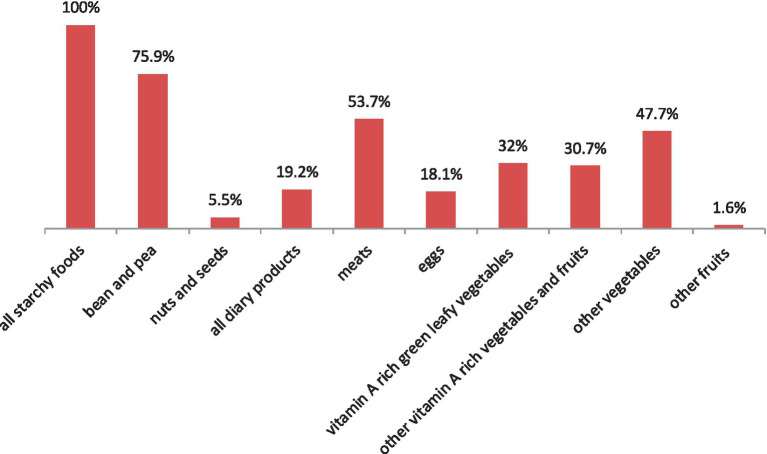
Consumption of food groups among lactating mothers in Northwest Ethiopia (*N* = 415).

The prevalence of undernutrition and low dietary diversity scores among respondents were 13.5% (95% CI: 10.4, 17.2) and 64.8% (95% CI: 60.0, 69.4), respectively. The nutritional status of lactating mothers for overweight and obese was 19.8 and 2.9%, respectively. The respondent’s median measurement of weight, height, and MUAC was 56.8 ± 11.4 (median ± IQR), 1.57 ± 0.08 m, and 26.5 ± 4.0, respectively (Table 2). Consumption of all starchy staples (porridge, bread, rice, injera, and pasta) was high among respondents, accounting for 100%. This was followed by beans and peas (mature beans or peas) and lentils (fresh or dried seed) (75.9%). The food groups that were least consumed by the respondents were nuts and seeds (5.5%) and other fruits (lemons and apples) (1.6%), respectively (Figure 1).

### Factors associated with the undernutrition of lactating mothers

In the bivariable binary logistic regression analysis, several variables showed a significant association (with a *p*-value ≤0.25) with the nutritional status of lactating mothers. These variables included age, educational and occupational status of lactating mothers, educational and occupational status of the husband, foods restricted in the community during the lactation period, nutritional knowledge, dietary diversity score, food security status, wealth index, and frequency of meals. Until now, in the multivariable binary logistic regression analysis, notable associations were observed between nutritional status and age, educational status of lactating mothers and their husbands, nutritional knowledge, dietary diversity, and meal frequency. Being of younger age was associated with a 2.30 times higher likelihood of being undernourished than the older individuals [AOR = 2.30, 95% CI (1.09, 5.43)]. The odds of becoming undernourished among lactating mothers with a low dietary diversity score were 2.26 times higher than those with a high dietary diversity score [AOR = 2.26, 95% CI (1.01,5.10)]. Respondents who had poor nutritional knowledge were 2.56 times more likely to become undernourished than those with good nutritional knowledge [AOR = 2.56, 95% CI (1.03, 6.51)]. The odds of experiencing undernutrition among lactating mothers with a meal frequency of ≤3 times per day were 4.06 times higher than ≥3 times per day [AOR = 4.06, 95% CI (0.71, 9.65)]. Respondents who completed primary school were 3.20 times more likely to be undernourished than those who had completed college or higher [AOR = 3.20, 95% CI (1.01, 9.11)]. The respondent’s husband, who completed secondary school, was 2.28 times more likely to be undernourished than those who completed college or higher [AOR = 2.28, 95% CI (1.25, 8.53)] (Table 3).

**Table 3:** Bivariable and multivariable binary logistic regression analyses of undernutrition among lactating mothers in Northwest Ethiopia (*N* = 415).

		Undernutrition			
Variables	Categories	Yes	No	COR (95% CI)	AOR (95% CI)
Age	20–30	45	229	2.32 (1.16, 4.65)**	2.30 (1.09, 5.43)**
	31–49	11	130	1	1
Educational status of lactating mothers	Cannot read and write	17	35	4.21 (1.97, 8.98)**	3.20 (1.01, 9.11)**
	Primary school	17	76	1.94 (0.95, 3.97)*	1.73 (0.69, 4.33)*
	Secondary school	4	92	0.38 (0.12, 1.15)*	0.29 (0.08, 1.02)*
	College and above	18	156	1	1
Educational status of the husband	Cannot read and write	9	16	5.84 (2.30, 14.82)**	1.78 (0.36, 8.76)
	Primary school	12	59	2.11 (0.98, 4.54)**	1.06 (0.32, 3.58)
	Secondary school	14	62	2.34 (1.13, 4.88)**	2.28 (1.25, 8.53)**
	College and above	21	218	1	1
Occupational status of the lactating mother	Housewife	20	143	1	1
	Government employee	9	100	0.64 (0.28, 1.47)	0.58 (0.27, 2.26)
	Private employee	10	45	1.59 (0.69, 3.64)	1.47 (0.55, 3.92)
	Merchant	5	44	0.81 (0.29, 2.29)	0.80 (0.26, 3.24)
	Daily laborers and others	12	27	3.18 (1.39, 7.25)**	1.62 (0.44, 5.91)
Occupational status of the husband	Merchant	7	83	1	1
	Government employee	22	153	1.71 (0.70, 4.16)*	1.59 (0.79, 7.29)*
	Private employee	12	92	1.55 (0.58, 4.11)	1.08 (0.35, 3.37)
	Daily laborers and others	15	31	5.74 (2.14, 15.40)**	2.19 (0.59, 8.15)*
Frequency of meals	≤3 meals per day	48	201	4.72 (2.17, 10.26)**	4.06 (0.71, 9.65)**
	>3 meals per day	8	158	1	1
Food restrictions in the community during lactation	No	50	338	0.52 (0.20, 1.35)*	0.45 (0.14, 1.46)*
	Yes	6	21	1	1
Nutritional knowledge status	Poor	31	113	2.94 (1.41, 6.14)**	2.56 (1.03,6.51)**
	Medium	14	128	1.17 (0.51, 2.69)	1.24 (0.49, 3.14)
	Good	11	118	1	1
Dietary diversity score (DDS)	High DDS	10	136	1	1
	Low DDS	46	223	2.81 (1.37, 5.74)**	2.26 (1.01,5.10)**
Food security status	Food secure	39	314	1	1
	Food insecure	17	45	3.04 (1.59, 5.82)**	0.42 (0.13, 1.44)*
Wealth index	Poorest	20	55	3.16 (1.47, 6.82)**	0.66 (0.22, 2.04)
	Poor	10	87	1.10 (0.42, 2.39)	0.93 (0.35, 2.46)
	Middle	4	47	0.74 (0.23, 2.39)	0.73 (0.20, 2.64)
	Rich	9	57	1.37 (0.55, 3.40)	1.35 (0.73, 5.79)*
	Richest	13	113	1	1

NB; COR: crude odds ratio, AOR: adjusted odds ratio, CI: confidence interval, *: *p*- value ≤ 0.25, **: *p*- value < 0.05.

### Factors associated with dietary diversity scores of lactating mothers

In the bivariable binary logistic regression analysis, several variables showed a significant association (with a *p*-value of ≤0.25) with the dietary diversity score of lactating mothers. These variables included age, child’s age, educational and occupational levels of lactating mothers, husband’s occupational status, foods restricted in the community during the lactation period, food security status, wealth index, and frequency of meals. However, in the multivariable binary logistic regression analysis, remarkable associations were observed between the dietary diversity score and age, food security, wealth index, and nutritional status. Respondents aged between 20 and 30 were 1.46 times more likely to have a low dietary diversity score than those aged between 31 and 45 [AOR = 1.46, 95% CI (1.01, 2.48)]. Respondents from food insecure households were 3.41 times more likely to have a low dietary diversity score than those from food secure households [AOR = 3.41, 95% CI (1.21, 9.63)]. Respondents from the poorest household were 2.31 times more likely to experience a low dietary diversity score than the richest household [AOR = 2.31, 95% CI (1.02, 5.32)]. However, the odds of a low dietary diversity score among respondents with a rich wealth index decreased by 50% compared to the richest [AOR = 0.50, 95% CI (0.27, 0.93)] (Table 4).

**Table 4:** Bivariable and multivariable binary logistic regression analyses of dietary diversity scores among lactating mothers in Northwest Ethiopia (*N* = 415).

		Dietary diversity score			
Variables	Categories	Low DDS	High DDS	COR (95% CI)	AOR (95% CI)
Age in years	20–30	186	88	1.48(0.97, 2.25)*	1.46(1.01, 2.48)**
	31–49	83	58	1	1
Child’s age	6 months to 1 year	136	84	0.75(0.50, 1.13)*	0.72(0.50, 1.20)*
	1–2	133	62	1	1
Educational status of lactating mothers	Cannot read and write	46	6	5.16(2.09, 12.73)**	0.93(0.27, 3.13)
	Primary school	59	34	1.17(0.70, 1.96)	0.61(0.30, 1.23)*
	Secondary school	60	36	1.12(0.67, 1.87)	1.12(0.63, 1.99)
	College and above	104	70	1	1
Occupational status of lactating mothers	Housewife	105	58	1	1
	Government employee	61	48	0.70(0.43, 1.15)*	0.97(0.51, 1.83)
	Private employee	36	19	1.05(0.55, 1.99)	1.03(0.49, 2.17)
	Merchant	30	19	0.87(0.45, 1.68)	1.20(0.56, 2.59)
	Daily laborers and others	37	2	10.22(2.38, 43.94)**	2.25(0.42, 12.18)
Occupational status of the husband	Merchant	54	36	1	1
	Government employee	100	75	0.89(0.53, 1.49)	0.70(0.38, 1.26)*
	Private employee	72	32	1.50(0.83, 2.71)*	1.15(0.61, 2.15)
	Daily laborer	43	3	9.56(2.75, 33.15)**	2.44(0.59, 10.02)*
Frequency of meals	≤3 meals per day	176	73	1.89(1.26, 2.85)**	1.10(0.69, 1.76)
	>3 meals per day	93	73	1	1
Food restrictions in the community during lactation	No	248	140	0.51(0.20, 1.28)*	0.47(0.24, 1.89)
	Yes	21	6	1	1
Food security status	Food secure	212	141	1	1
	Food insecure	57	5	7.58(2.97, 19.38)**	3.41(1.21, 9.63)**
Wealth index	Poorest	66	9	4.07(1.86, 8.94)**	2.31(1.02, 5.32)**
	Poor	59	38	0.86(0.50, 1.49)	0.87(0.50, 1.51)
	Middle	31	20	0.86(0.44, 1.68)	0.89(45, 1.77)
	Rich	32	34	0.52(0.29, 0.96)**	0.50(0.27, 0.93)**
	Richest	81	45	1	1

NB; COR, crude odds ratio; AOR, adjusted odds ratio; CI, confidence interval, *: *p*- value ≤ 0.25, **: *p*- value < 0.05.

## Discussion

The prevalence of undernutrition in the study area was 13.5%. In a study conducted in the Wolayita Zone, SNNPRs (15.8%) reported findings similar to those of the current study (40). In contrast to the findings of the current study, most Ethiopian studies reported higher prevalence in rural Yilmana Densa woreda (22.6%) (41), Wombera district (25.4%) (24), Debre Tabor (17.9%) (42), Dessie town (21%) (43), Sekota (54.8%) (27), the pastoral community of the Afar region (32.8%) (44), rural Tigray (50.6%) (25), Samer Worda, south eastern Tigray (31%) (45), Chiro district, eastern Ethiopia (30.7%) (46), Ambo district (21.5%) (47), Dire Dawa (22%) (48), Borena Zone, Southern Ethiopia (17.7%) (49), and Nekemte (20%) (27). Furthermore, my result was higher than those of the study conducted in the Bench Sheko district, in southwest Ethiopia (10.3%) (50). The disparity might be attributed to the study population’s diverse sociodemographic and socioeconomic characteristics.

The prevalence of a low dietary diversity score among respondents in this area was 64.8%. This finding was consistent with that of the study conducted in Gambela town (29). On the other hand, higher results were reported in Finote Selam district (76.9%) (26), Gedeo Zone, southern Ethiopia (75.5%) (28), and Abala district, Afar region (76.4%) (51). In addition, the result of the current study was higher than the findings from Worta town, South Gondar Zone (56.9%) (52), Dessie town (55.6%) (43), and Aksum Town (56.4%) (53). These similarities and differences may be attributed to differences in socioeconomic level, sample size, seasonal variation, and research setting. Cereals and legumes are the most common products in the study region, followed by fruits and vegetables. This might contribute to the low consumption of foods that are least required for dietary diversification. Furthermore, the gap might be attributed to discrepancies in dietary variety measuring methodologies; the current study used the new WHO-suggested indicator that includes 10 food categories, whereas previous studies used indicators based on 9 food groups.

Age was a significant factor in undernutrition. Younger respondents were more likely to become undernourished than older respondents. This finding was supported by studies conducted in Dessie town (43), Raya, Alamata (54), Rural Ambo District, Oromia (47), and Dire Dawa (48). This might be related to the poor socioeconomic status of lactating mothers, which could be threatened by a negative alteration in marital status.

Another factor affecting nutritional status was the dietary diversity score. The odds of undernutrition among lactating mothers with a low dietary diversity score were higher than those with a high dietary diversity score. The current study was in line with studies conducted in Dessie town (43), Dire Dawa (48), Afar region (44), Rural Tigray (25), Eastern, Ethiopia (48), Borena Zone, and Southern Ethiopia (17.7%) (49). The low dietary diversity score of the respondents may be due to a lower frequency of meals per day, a lack of extra meals during the lactation period, and a lack of knowledge regarding diversified food consumption. Human health and wellbeing depend heavily on nutrition and food. A nutritious diet is necessary for appropriate growth, development, and maintenance at all stages of life. The quantity and quality of food consumed substantially influence overall health. A diverse diet indicates that necessary nutrients are being consumed in suitable amounts. Several studies have found that increased dietary diversity relates to greater health and a lower risk of different chronic illnesses.

Furthermore, nutritional knowledge was a significant variable for the undernutrition of lactating mothers. Respondents with poor nutritional knowledge were more likely to become undernourished than those having good nutritional knowledge. Similar results were reported in Womberma District, Northwest Ethiopia (24), Burie Town, Northwest Ethiopia (55), and Nekemte (56). This may be because such educational initiatives have a favorable influence on the health and wellbeing of communities.

Moreover, meal frequency was a factor affecting undernutrition. The odds of experiencing undernutrition among lactating mothers with a meal frequency of less or equal to 3 times per day was higher among respondents having a meal frequency of greater than 3 times. This result is supported by studies conducted by Debre Tabor (42), and Raya, Alamata (54). This finding revealed that women who ate fewer meals per day had a higher risk of lower weight. One possible explanation for this tendency is that these people consume fewer calories as a result of fewer meals.

Finally, the educational status of the respondents and their husbands was significantly associated with undernutrition. Respondents and their husbands who completed primary school were more likely to be undernourished than those who completed college and above. Parallel findings were conducted in Burie town, Northwest Ethiopia (55), Abala district, Afar region (51), southern Ethiopia (28), Dire Dawa (48), Rural Ambo District, Oromia (47), Eastern, Ethiopia (48), and Wolayita Zone, SNNPRs (40). One possible explanation is that people with formal education are more likely to receive nutritional information and absorb educational messages conveyed through various media channels. This increased exposure can enhance their understanding of dietary diversity, promote healthy eating, and lead to long-term behavioral changes in their health status.

Respondents aged between 20 and 30 years were more likely to have a low dietary diversity score than those aged between 31 and 45 years. Concurrent findings were reported in Debre Tabor (57), Dessie town (43), Gedeo Zone (28), and Bangladesh (58). Respondents from food insecure households were more likely to have low dietary diversity than those from food secure households. The same results were reported in Dessie town (43), Ataye District, North Shoa Zone (31), Worta town, South Gondar Zone (52), Finote Selam District, Northwest Ethiopia (26), Lay Gayint District, South Gondar Zone (59), Pawie district, Northwest Ethiopia (60), Nepal (61), Angecha districts, and Southern Ethiopia (62). This could be clarified as follows: Food security supports the consumption of sufficient amounts of food and quality of diet, which contributes to a higher dietary variety score and a good nutritional status.

Respondents from the poorest households were more likely to experience a low dietary diversity score than those from the richest. However, the odds of low dietary diversity among respondents with a rich wealth index decreased by 50% compared to the richest. This result is consistent with studies conducted in Finote Selam District, Northwest Ethiopia (26), and Ataye District, North Shoa Zone (31). A possible justification for this similarity might be their economic status. Dietary diversity is highly associated with a family’s income and socioeconomic position. Individuals with higher socioeconomic status consumed more high-quality food diversity than those with lower socioeconomic status.

## Conclusion and recommendations

The prevalence of undernutrition among respondents in the current study area was higher than that reported in the EDHS Seated guidelines. Similarly, the prevalence of low dietary diversity scores was high. Age, educational status of lactating mothers and their husbands, nutritional knowledge, dietary diversity score, and meal frequency were significant factors associated with the nutritional status of lactating mothers. Furthermore, in the multivariable binary logistic regression analysis, age, food security, and wealth index were significant factors for the dietary diversity score of lactating mothers. Lactating mothers’ nutritional status did not meet the national or international standards. As a result, lactating mothers, their families, and communities should receive ongoing nutrition education to promote food intake and adequate dietary information throughout lactation to increase the health and nutrition benefits of lactating mothers and their children.

## Limitations and strengths of the study

The current study is cross-sectional, meaning it cannot establish cause–effect relationships. Additionally, seasonal variations in food consumption may influence the results, as dietary information was collected only for the specific season in which the study was conducted. The reliance on a single 24-h dietary recall may not fully represent the respondents’ usual dietary practices. Furthermore, recall bias and social desirability bias are the study’s limitations. However, the study’s strengths were the use of the WHO-recommended 10 dietary categories and the exclusion of festival days.

## Data Availability

The original contributions presented in the study are included in the article/supplementary material, further inquiries can be directed to the corresponding author/s.

## References

[1] Mardani M Abbasnezhad A Ebrahimzadeh F Roosta S Rezapour M Choghakhori R. Assessment of nutritional status and related factors of lactating women in the urban and rural areas of southwestern Iran: a population-based cross-sectional study. Int J Community Based Nurs Midwifery. (2020) 8:73–83. doi: 10.30476/ijcbnm.2019.73924.0 32039281 PMC6969947

[2] Maqbool M Dar MA Gani I Mir SA Khan M Bhat AU. Maternal health and nutrition in pregnancy: an insight. World J Pharm Pharm Sci. (2019) 8:450–9. doi: 10.20959/wjpps20193-13290

[3] Marshall NE Abrams B Barbour LA Catalano P Christian P Friedman JE. The importance of nutrition in pregnancy and lactation: lifelong consequences. Am J Obstet Gynecol. (2022) 226:607–32. doi: 10.1016/j.ajog.2021.12.035 34968458 PMC9182711

[4] Kominiarek MA Rajan P. Nutrition recommendations in pregnancy and lactation. Med Clin North Am. (2016) 100:1199–215. doi: 10.1016/j.mcna.2016.06.004, PMID: 27745590 PMC5104202

[5] Kennedy G Lee WTK Termote C Charrondiere R Yen J Tung A. Guidelines on assessing biodiverse foods in dietary intake surveys. Rome (Italy): FAO (2017). 96 p.

[6] Marangoni F Cetin I Verduci E Canzone G Giovannini M Scollo P. Maternal diet and nutrient requirements in pregnancy and breastfeeding. An Italian consensus document. Nutrients. (2016) 8:8100629. doi: 10.3390/nu8100629 PMC5084016 27754423

[7] Haque S Salman M Rahman MS Rahim A Hoque MN. Mothers' dietary diversity and associated factors in megacity Dhaka, Bangladesh. Heliyon. (2023) 9:e19117. doi: 10.1016/j.heliyon.2023.e19117 37636472 PMC10450986

[8] Huang M Sudfeld C Ismail A Vuai S Ntwenya J Mwanyika-Sando M. Maternal dietary diversity and growth of children under 24 months of age in rural Dodoma. Tanzania Food Nutr Bull. (2018) 39:219–30. doi: 10.1177/0379572118761682 29562752

[9] Black RE Allen LH Bhutta ZA Caulfield LE de Onis M Ezzati M. Maternal and child undernutrition: global and regional exposures and health consequences. Lancet. (2008) 371:243–60. doi: 10.1016/S0140-6736(07)61690-0, PMID: 18207566

[10] Christian P Smith ER Zaidi A. Addressing inequities in the global burden of maternal undernutrition: the role of targeting. BMJ Glob Health. (2020) 5:e002186. doi: 10.1136/bmjgh-2019-002186 PMC7101036 32231793

[11] Seifu BL Mare KU Legesse BT Tebeje TM. Double burden of malnutrition and associated factors among women of reproductive age in sub-Saharan Africa: a multilevel multinomial logistic regression analysis. BMJ Open. (2024) 14:e073447. doi: 10.1136/bmjopen-2023-073447, PMID: 38341217 PMC10862289

[12] Girma B Nigussie J Molla A Mareg M. Under-nutrition and associated factors among lactating mothers in Ethiopia: a systematic review and Meta-analysis. Matern Child Health J. (2022) 26:2210–20. doi: 10.1007/s10995-022-03467-6 36040618

[13] Bitew ZW Alemu A Ayele EG Worku T. Dietary diversity and practice of pregnant and lactating women in Ethiopia: a systematic review and meta-analysis. Food Sci Nutr. (2021) 9:2686–702. doi: 10.1002/fsn3.2228, PMID: 34026082 PMC8116864

[14] Young MF Ramakrishnan U. Maternal undernutrition before and during pregnancy and offspring health and development. Ann Nutr Metab. (2021) 1:1–13. doi: 10.1159/000510595, PMID: 33524980

[15] Müller O Jahn A. Malnutrition and maternal and child health In: Ehiri J, editor. Maternal and Child Health: Global Challenges, Programs, and Policies. Berlin: Springer (2009). 287–310.

[16] Triunfo S Lanzone A. Impact of maternal under nutrition on obstetric outcomes. J Endocrinol Investig. (2015) 38:31–8. doi: 10.1007/s40618-014-0168-4 25194427

[17] Mason JB Saldanha LS Martorell R. The importance of maternal undernutrition for maternal, neonatal, and child health outcomes: an editorial. Food Nutr Bull. (2012) 33:S3–5. doi: 10.1177/15648265120332S101, PMID: 22913104

[18] Dammann KW Smith C. Factors affecting low-income women's food choices and the perceived impact of dietary intake and socioeconomic status on their health and weight. J Nutr Educ Behav. (2009) 41:242–53. doi: 10.1016/j.jneb.2008.07.003 19508929

[19] Nguyen PH Kachwaha S Tran LM Sanghvi T Ghosh S Kulkarni B. Maternal diets in India: gaps, barriers, and opportunities. Nutrients. (2021) 13:3103534. doi: 10.3390/nu13103534, PMID: 34684535 PMC8540854

[20] Erzse A Desmond C Hofman K Barker M Christofides NJ. Qualitative exploration of the constraints on mothers' and pregnant women's ability to turn available services into nutrition benefits in a low-resource urban setting, South Africa. BMJ Open. (2023) 13:e073716. doi: 10.1136/bmjopen-2023-073716, PMID: 37993159 PMC10668265

[21] World Health Organization. WHO guidelines approved by the guidelines review committee. WHO recommendations on health promotion interventions for maternal and newborn health. Geneva: World Health Organization (2015). 26180864

[22] Althabe F Bergel E Cafferata ML Gibbons L Ciapponi A Alemán A. Strategies for improving the quality of health care in maternal and child health in low- and middle-income countries: an overview of systematic reviews. Paediatr Perinat Epidemiol. (2008) 22:42–60. doi: 10.1111/j.1365-3016.2007.00912.x 18237352

[23] Gil JDB Reidsma P Giller K Todman L Whitmore A van Ittersum M. Sustainable development goal 2: improved targets and indicators for agriculture and food security. Ambio. (2019) 48:685–98. doi: 10.1007/s13280-018-1101-4, PMID: 30267284 PMC6509081

[24] Berihun S Kassa GM Teshome M. Factors associated with underweight among lactating women in Womberma woreda, Northwest Ethiopia; a cross-sectional study. BMC Nutr. (2017) 3:46. doi: 10.1186/s40795-017-0165-z 32153826 PMC7050864

[25] Desalegn BB Lambert C Riedel S Negese T Biesalski HK. Ethiopian orthodox fasting and lactating mothers: longitudinal study on dietary pattern and nutritional status in rural Tigray, Ethiopia. Int J Environ Res Public Health. (2018) 15. doi: 10.3390/ijerph15081767 PMC6121597 30126089

[26] Gebrie YF Dessie TM. Bayesian analysis of dietary diversity among lactating mothers in Finote Selam District, Northwest Ethiopia: a cross-sectional study. Biomed Res Int. (2021) 2021:9604394. doi: 10.1155/2021/9604394 34497855 PMC8421177

[27] Mengstie MA Worke MD Belay Y Chekol Abebe E Asmamaw Dejenie T Abdu Seid M. Undernutrition and associated factors among internally displaced lactating mothers in Sekota camps, northern Ethiopia: a cross-sectional study. Front Nutr. (2023) 10:1108233. doi: 10.3389/fnut.2023.1108233 36866050 PMC9971014

[28] Molla W Mengistu N Madoro D Assefa DG Zeleke ED Tilahun R. Dietary diversity and associated factors among lactating women in Ethiopia: cross sectional study. Int J Afr Nurs Sci. (2022) 17:100450. doi: 10.1016/j.ijans.2022.100450

[29] Teferi T Endalk G Ayenew GM Fentahun N. Inadequate dietary diversity practices and associated factors among postpartum mothers in Gambella town, Southwest Ethiopia. Sci Rep. (2023) 13:7252. doi: 10.1038/s41598-023-29962-6 37142603 PMC10160103

[30] Administration Total Population Estimation. *2006 - 2019 Bahir Dar City Administration total population estimation; Bahir Dar City administration office*. 2020 Annual report unpublished. (2020).

[31] Getacher L Egata G Alemayehu T Bante A Molla A. Minimum dietary diversity and associated factors among lactating mothers in Ataye District, North Shoa zone, Central Ethiopia: a community-based cross-sectional study. J Nutr Metab. (2020) 2020:1823697. doi: 10.1155/2020/1823697, PMID: 33520304 PMC7817227

[32] Salvador Castell G Pérez Rodrigo C Ngo de la Cruz J Aranceta BJ. Household food insecurity access scale (HFIAS). Nutr Hosp. (2015) 31:272–8. doi: 10.3305/nh.2015.31.sup3.8775 25719795

[33] Coates J Swindale A Bilinsky P. *Household Food Insecurity Access Scale (HFIAS) for measurement of food access: indicator guide: version 3*. (2007).

[34] Mengie GM Worku T Nana A. Nutritional knowledge, dietary practice and associated factors among adults on antiretroviral therapy in Felege Hiwot referral hospital, Northwest Ethiopia. BMC Nutr. (2018) 4:46. doi: 10.1186/s40795-018-0256-5 32153907 PMC7050901

[35] Italy N Division CP Thompson B Amoroso L Agriculture, Department CP. *Measurement of dietary diversity for monitoring the impact of food-based approaches. Improving diets and nutrition: Food-based Approaches: CABI Wallingford UK*. pp. 284–290. (2014).

[36] Phillips W Doley J Boi K. Malnutrition definitions in clinical practice: to be E43 or not to be? Health Inf Manag. (2020) 49:74–9. doi: 10.1177/1833358319852304, PMID: 31130015

[37] Gezimu GG. Intra-household decision-making and their effects on women dietary diversity: evidence from Ethiopia. Ecol Food Nutr. (2022) 61:705–27. doi: 10.1080/03670244.2022.2135509 36256907

[38] Woolley K Fishbach A Wang RM. Food restriction and the experience of social isolation. J Pers Soc Psychol. (2020) 119:657–71. doi: 10.1037/pspi0000223 31724417

[39] Conde WL Silva IVD Ferraz FR. Undernutrition and obesity trends in Brazilian adults from 1975 to 2019 and its associated factors. Cad Saude Publica. (2022) 38. doi: 10.1590/0102-311Xe00149721 35613255

[40] Julla BW Haile A Mersha GA Eshetu S Kuche D Asefa T. Chronic energy deficiency and associated factors among lactating mothers (15-49 years old) in Offa Woreda, Wolayita zone, SNNPRs, Ethiopia. World Sci Res. (2018) 5:13–23. doi: 10.20448/journal.510.2018.51.13.23

[41] Wubetie BY Mekonen TK. Undernutrition and associated factors among lactating mothers in rural Yilmana Densa District, Northwest Ethiopia: a community-based cross-sectional study. Food Sci Nutr. (2023) 11:1383–93. doi: 10.1002/fsn3.3176, PMID: 36911817 PMC10002878

[42] Engidaw MT Gebremariam AD Tiruneh SA Asnakew DT Abate BA. Chronic energy deficiency and its associated factors among lactating women in Debre Tabor general hospital, northcentral Ethiopia. J Fam Med Healthc. (2019) 5:1–7. doi: 10.11648/j.jfmhc.20190501.11

[43] Seid A Cherie HA. Dietary diversity, nutritional status and associated factors among lactating mothers visiting government health facilities at Dessie town, Amhara region, Ethiopia. PLoS One. (2022) 17:e0263957. doi: 10.1371/journal.pone.0263957, PMID: 35176095 PMC8853554

[44] Mulaw GF Mare KU Anbesu EW. Nearly one-third of lactating mothers are suffering from undernutrition in pastoral community, Afar region, Ethiopia: community-based cross-sectional study. PLoS One. (2021) 16:e0254075. doi: 10.1371/journal.pone.0254075, PMID: 34242276 PMC8270417

[45] Haileslassie K Mulugeta A Girma M. Feeding practices, nutritional status and associated factors of lactating women in Samre Woreda, south eastern zone of Tigray, Ethiopia. Nutr J. (2013) 12:28. doi: 10.1186/1475-2891-12-28, PMID: 23452646 PMC3599359

[46] Minas S Ayele BH Sisay M Fage SG. Undernutrition among khat-chewer and non-chewer lactating women in chiro district, eastern Ethiopia: comparative cross-sectional study. SAGE Open Med. (2022) 10:20503121221100143. doi: 10.1177/20503121221100143, PMID: 35646352 PMC9136449

[47] Zerihun E Egata G Mesfin F. Under nutrition and its associated factors among lactating mothers in rural ambo district, west Shewa zone, Oromia region, Ethiopia. East Afr J Health Biomed Sci. (2016) 1:39–48.

[48] Tariku Z Tefera B Samuel S Derese T Markos M Dessu S. Nutritional status and associated factors among lactating women in Dire Dawa, Ethiopia. J Obstet Gynaecol Res. (2022) 48:1183–92. doi: 10.1111/jog.15198 35194884

[49] Bekele H Jima GH Regesu AH. Undernutrition and associated factors among lactating women: community-based cross-sectional study in Moyale District, Borena zone, Southern Ethiopia. Adv Public Health. (2020) 2020:4367145. doi: 10.1155/2020/4367145

[50] Sebeta A Girma A Kidane R Tekalign E Tamiru D. Nutritional status of postpartum mothers and associated risk factors in Shey-Bench District, bench-Sheko zone, Southwest Ethiopia: a community based cross-sectional study. Nutr Metab Insights. (2022) 15:1088243. doi: 10.1177/11786388221088243, PMID: 35493421 PMC9044780

[51] Mulaw GF Feleke FW Mare KU. Only one in four lactating mothers met the minimum dietary diversity score in the pastoral community, Afar region, Ethiopia: a community-based cross-sectional study. J Nutr Sci. (2021) 10:e41. doi: 10.1017/jns.2021.28, PMID: 34164120 PMC8190715

[52] Nigussie B. Dietary diversity and associated factors among lactating women in woreta town. Ethiopia: South Gondar Zone (2020).

[53] Weldehaweria NB Misgina KH Weldu MG Gebregiorgis YS Gebrezgi BH Zewdie SW. Dietary diversity and related factors among lactating women visiting public health facilities in Aksum town, Tigray, Northern Ethiopia. BMC Nutr. (2016) 2:1–9. doi: 10.1186/s40795-016-0077-357

[54] Sitotaw IK Hailesslasie K Adama Y. Comparison of nutritional status and associated factors of lactating women between lowland and highland communities of district Raya, Alamata, southern Tigiray, Ethiopia. BMC Nutr. (2017) 3:61. doi: 10.1186/s40795-017-0179-6 32153841 PMC7050921

[55] Sewalem MA. *Assesment of nutritional status and associated factors among lactating mothers in burie town, North West Ethiopa*. (2022).

[56] Hundera T Fekadu Gemede H Wirtu D Keneie D. *Nutritional status and associated factors among lactating mothers in Nekemte referral hospital and health centers, Ethiopia*, p. 35. (2015).

[57] Engidaw MT Gebremariam AD Tiruneh SA Asnakew DT Abate BA. Dietary diversity and associated factors among lactating mothers in Debre Tabor general hospital, northcentral Ethiopia. Int J. (2019) 5:17. doi: 10.18203/issn.2454-2156.IntJSciRep20185350

[58] Shaun MMA Nizum MWR Shuvo MA Fayeza F Faruk MO Alam MF. Determinants of minimum dietary diversity of lactating mothers in rural northern region of Bangladesh: a community-based cross-sectional study. Heliyon. (2023) 9:e12776. doi: 10.1016/j.heliyon.2022.e12776 36632115 PMC9826838

[59] Fentahun N Alemu E. Nearly one in three lactating mothers is suffering from inadequate dietary diversity in Amhara region, Northwest Ethiopia. J Nutr Metab. (2020) 2020:7429034. doi: 10.1155/2020/7429034, PMID: 33029395 PMC7530474

[60] Mulatu S Dinku H Yenew C. Dietary diversity (DD) and associated factors among lactating women (LW) in Pawie district, northwest, Ethiopia, 2019: community-based cross-sectional study. Heliyon. (2021) 7:e08495. doi: 10.1016/j.heliyon.2021.e08495, PMID: 34917799 PMC8645438

[61] Singh DR Ghimire S Upadhayay SR Singh S Ghimire U. Food insecurity and dietary diversity among lactating mothers in the urban municipality in the mountains of Nepal. PLoS One. (2020) 15:e0227873. doi: 10.1371/journal.pone.0227873, PMID: 31935272 PMC6959598

[62] Boke MM Geremew AB. Low dietary diversity and associated factors among lactating mothers in Angecha districts, southern Ethiopia: community based cross-sectional study. BMC Res Notes. (2018) 11:892. doi: 10.1186/s13104-018-4001-6 30547839 PMC6295037

